# A multicenter-validated interpretable transformer model for pituitary microadenoma detection on non-contrast multiparametric MRI

**DOI:** 10.1186/s12880-026-02391-3

**Published:** 2026-05-02

**Authors:** Siru Kang, Wenxia Yang, Yijun Yu, Kai Wang, Wenhuan Yuan, Yanli Jiang, Jing Zhang

**Affiliations:** 1 Department of Magnetic Resonance, The Second Hospital & Clinical Medical School, Lanzhou University, No. 82 Cuiyingmen, Chengguan District, Lanzhou, 730030 China; 2 Gansu Province Clinical Research Center for Functional and Molecular Imaging, Lanzhou, China; 3 Gansu Medical MRI Equipment Application Industry Technology Center, Lanzhou, China; 4 Lincang People’s Hospital, Lincang, China; 5 Xiaogan Central Hospital, Xiaogan, China

**Keywords:** Convolutional neural network (CNN), Pituitary microadenomas, Explainable AI (XAI), SHAP, Grad-CAM

## Abstract

**Background:**

Detecting pituitary microadenomas using non-contrast multi-parametric magnetic resonance imaging (MRI) is challenging yet essential for clinical decisions. This study aimed to develop a transformer deep learning (DL) model for detecting pituitary microadenomas based on non-contrast multiparametric MRI and explore the explainability techniques to enhance transparency in convolutional neural network (CNN)-based classification. The primary research question addressed is how to improve the accuracy, generalization, and interpretability of CNNs for microadenomas detection.

**Methods:**

Non-contrast multiparametric MRI sella area scans of 590 patients were retrospectively collected from three hospitals. The development and comparison of 2D_DL, 2.5D_DL, 2D_multichannel, and transformer models for classification. By incorporating Explainable AI (XAI), including Gradient-weighted Class Activation Mapping(Grad-CAM) and SHapley Additive exPlanations (SHAP), we improve model interpretability.

**Results:**

The performance of the 2D_multichannel model, with an area under the curve (AUC) of 0.893, was better to that of the 2D_T1SAG_DL, 2D_T1COR_DL, 2D_T2COR_DL (AUC, 0.884, 0.779, and 0.846, respectively). The performance of the transformer model, with an area under the curve (AUC) of 0.985, was superior to that of the 2.5D_T1SAG_DL, 2.5D_T1COR_DL, 2.5D_T2COR_DL (AUC, 0.763, 0.863, and 0.835, respectively). The non-contrast MRI-based 2.5D_DL transformer model all shows outperforming performance in the internal and two external test sets (AUC, 0.874, 0.829, and 0.819, respectively).

**Conclusions:**

Given its robust diagnostic performance and enhanced interpretability, this model demonstrates significant potential for clinical translation as a decision-support tool in the detection of pituitary microadenomas.

**Supplementary Information:**

The online version contains supplementary material available at 10.1186/s12880-026-02391-3.

## Background

Pituitary neuroendocrine tumors (PitNETs) represent a diverse group of neoplasms that originate from the endocrine cells of the pituitary gland, and they are the most common tumor within the sellar and parasellar regions, accounting for 17.8% of nervous system tumors [1–3]. PitNETs are conventionally classified by size into microadenomas (maximum diameter < 1 cm) and macroadenomas (≥ 1 cm). While microadenomas have a high reported incidence on MRI in the general population (10–38%) [4] and are found in approximately 10% of healthy subjects as incidental findings [5]. Account of microadenomas’ smaller dimension or its signals appear normal, radiologists often missed in diagnosis when judged only based on conventional non-contrast multiparametric MR [6–8]. Just as was indicated in previous studies, Bashari et al. [9]. report that the normal anterior pituitary is isointense with gray matter on T1W/T2W imaging which contributes to the difficulty in detection of microadenoma in the absence of contrast, and the T1-weighted detection rate was about 55.7%, and Farabola et al. [10]. reported that T1W+T2W spinecho combined sequences were only able to identify 64% of microadenomas. To improve diagnostic accuracy, dynamic contrast-enhanced magnetic resonance imaging (DCE-MRI) has been used as a gold standard [11–13], it tends to increase the detection rates for microadenomas that are generally not visible on conventional non-contrast MRI images. However, recent data on MRI monitoring of pituitary tumors showed an accumulation of gadolinium in brain tissue, so reducing unnecessary scans is advised [4, 14]. This clinical dilemma necessitates strategies to balance diagnostic accuracy with the judicious use of contrast media.

Artificial intelligence (AI), particularly deep learning, has emerged as a powerful tool to address such challenges by extracting subtle patterns from imaging data [15]. Convolutional neural networks (CNNs), with their innate ability to autonomously learn hierarchical features from pixel data, are well-suited for identifying subtle lesions like microadenomas that may evade routine visual assessment [16–21]. These models have gained widespread recognition for their ability to process large-scale datasets efficiently, offering a scalable solution to the growing demand for high-precision medical imaging workflows [22–24]. However, CNNs are often considered “black-box” models due to limited interpretability, which poses a critical concern in clinical applications. To bridge this gap, explainable AI (XAI) techniques such as Grad-CAM (Gradient-weighted Class Activation Mapping) and SHapley Additive exPlanations (SHAP) has been employed to visualize regions contributing to model predictions [25, 26]. Integrating Grad-CAM with non-contrast ResNet architectures improves both the reliability and transparency of AI-driven microadenomas detection.

More recently, the Transformer architecture has emerged as a promising alternative due to its specific strengths relevant to our diagnostic task. Unlike CNNs, which excel at capturing local patterns, the self-attention mechanism at the core of Transformers [27, 28] is designed to model relationships across all elements of an input sequence. In the context of our 2.5D multi-slice MRI data, this translates to an ability to integrate contextual information across different anatomical slices and imaging sequences (T1, T2), effectively capturing long-range spatial and contrast dependencies. This capability is particularly advantageous for identifying the diffuse or heterogeneous signal patterns that can characterize microadenomas [29, 30]. Building upon previous efforts to apply Transformer-based multimodal fusion models in medicine [30, 31], our study presents a significant advancement in this domain.

Despite these individual advancements, a systematic comparison of different deep learning architectures and input strategies for pituitary microadenoma detection on non-contrast MRI remains lacking. Therefore, this study aims to fill this gap by: (1) to develop predictive models that can accurately identify the presence of pituitary microadenomas based on imaging characteristics derived from non-contrast multiparametric MRI; (2) we aimed to systematically compare the effectiveness of different imaging sequence combinations and deep learning architectures for this task. ⑶ to utilize model visualization techniques to elucidate the decision-making process of deep learning models, thereby assessing their potential as clinical decision support tools for improving microadenoma detection on non-contrast MRI. Through these efforts, we aspire to contribute to the ongoing advancement of diagnostic strategies for pituitary disorders and to lay the groundwork for future research aimed at optimizing patient management in this area.

## Methods

### Study participants

**Fig. 1:**
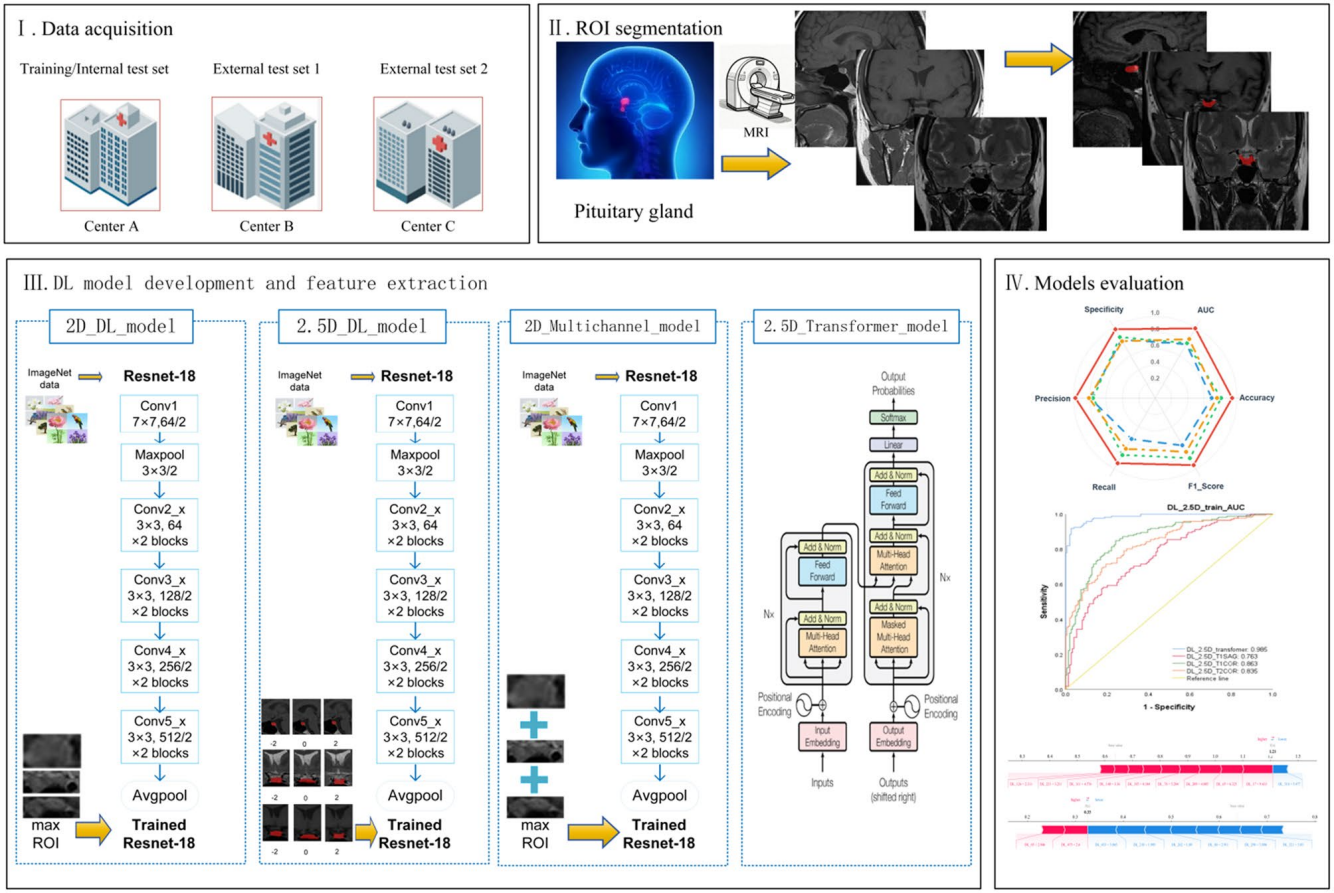
The research flowchart

**Fig. 2:**
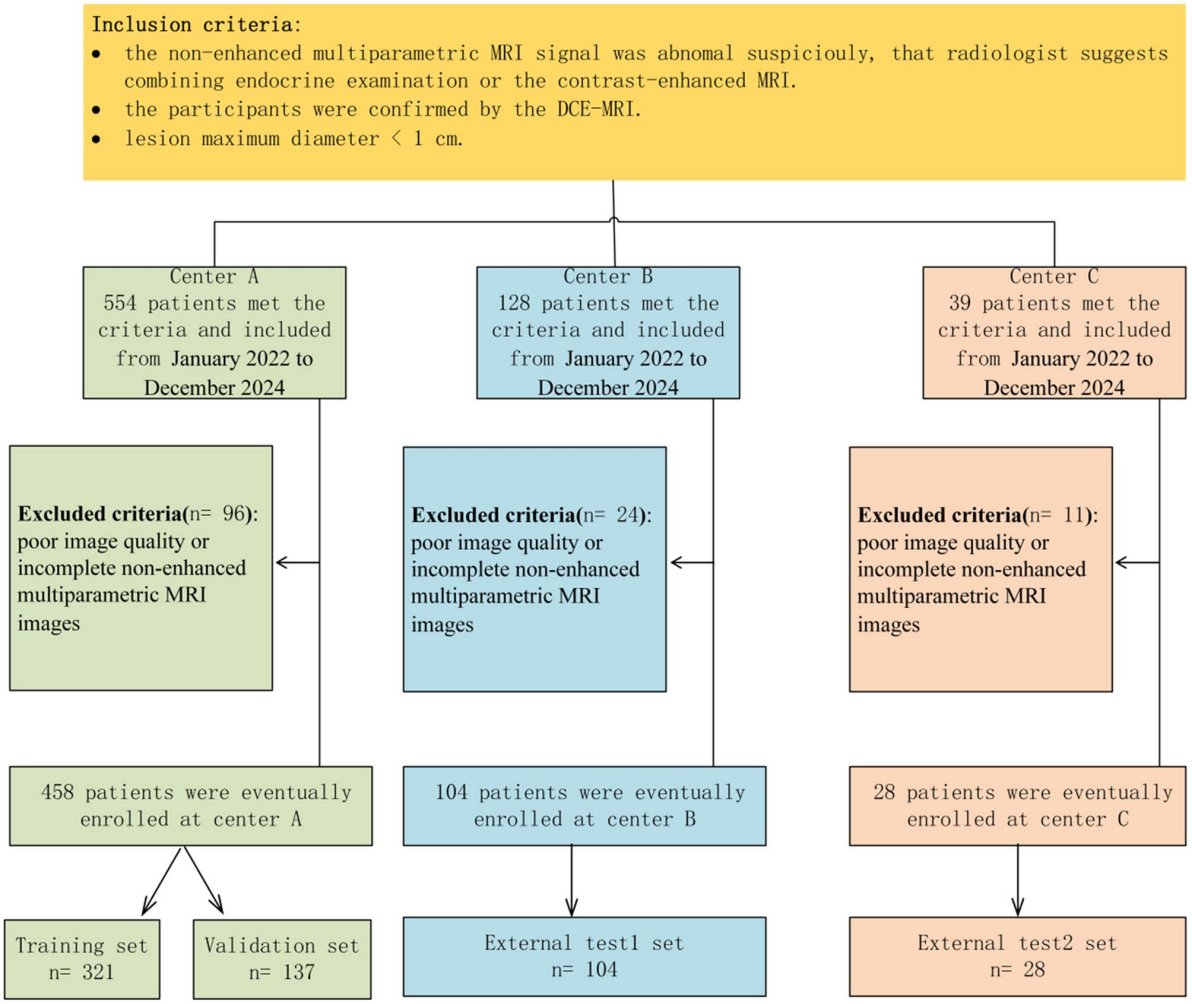
The flowchart of patient inclusion and exclusion in this study

The research flowchart is illustrated in Fig. 1. This study retrospectively collected 590 patients from the three hospitals from January 2022 to December 2024. Inclusion criteria: (1) the non-enhanced multiparametric MRI signal was abnormal suspiciouly, that radiologist suggests combining endocrine examination or the contrast-enhanced MRI, (2) participant status (microadenoma vs. normal) was confirmed by DCE-MRI, (3) lesion maximum diameter < 1 cm. Excluded criteria: poor image quality or incomplete non-enhanced multiparametric MRI images(Fig. 2).

Ethics: the Medical Ethics Committee approved this study (No. 2025 A-1222), which adhered to the principles of the Declaration of Helsinki. As a retrospective study without any adverse effect on de-identified subjects, it was exempt from the requirement for patient consent.

### Image acquisition

All participants underwent MRI scanning, three different MRI scanners (Verio, Aera, and Avanto, Siemens Healthcare) were used in this study, the scanning parameter details are illustrated in Table S1. The MR images were collected from the picture archiving and communication system (PACS) and stored in a workstation as digital imaging and communications in medicine (DICOM) for annotation and further analysis. The MRI images of all patients were resampled to 1.0*1.0*1.0 mm and N4 bias field [32] correction was used to adjust the image intensity to improve the image intensity inhomogeneity. To align the information from the two coronal position sequence MRIs, image registration was performed by registering T1W to T2W images using ITK-SNAP (V3.8.0).

### ROI segmentation and processing

ROIs were manually segmented using ITK-SNAP (version 3.6.0) by radiologists, each with 10 and 14 years of clinical experience, the doctor was blinded to diagnosis during the process of ROIs annotation. The ROIs in the MRI image was generated layer by layer along the boundary of the entire pituitary gland. To assess inter-rater reliability, 30 MR images were randomly selected and independently evaluated by two radiologists. Analysis of these evaluations yielded an intraclass correlation coefficient (ICC) of greater than 0.75, indicating good reliability. All the slices containing pituitary in the MRI sequences were formed as the input sequence of the deep learning model.

The key issue in identifying the ROI in the raw three-dimensional (3D) image dataset is to pinpoint the largest cross-sectional area, is known as a two-dimensional (2D) imaging technique. The selected range for these slices was 0, ± 2 from the central slice, resulting in a collection of 2D images (including three slices per patient) that were centered around the primary cross-sectional ROI area. The method, engineered to simulate 3D structural information, is known as a 2.5-dimensional (2.5D) imaging technique.

Typically, the 2.5D CNN-like ResNet takes RGB images as input. However, the 2D MRI images generated in each modality are grayscale with a single channel and cannot be directly processed by CNN models pre-trained on RGB images (Fig. S1). Therefore, we experimented with two different preprocessing methods to convert the MRI images into a format suitable for CNN input, in order to fully leverage the pre-trained networks. In brief, the MRI images were resized to 224 × 224, and then converted to RGB format using one of the following two methods.

1. the same MRI sequence (three slices per patient) served as reiterated input of RGB channels.
2. In the 2D multichannel paradigm, compatible modalities were combined, such as T1SAG, T2COR and T1COR, that served as the channel input of individual RGB channels.

### Data preparation and training settings

Before the deep learning model building, the enrolled patients were randomly divided into training and internal validation cohorts by a ratio of 7: 3 (Fig. 2). Our model development employed a transfer learning strategy, leveraging the representational power of ResNet architectures (ResNet18, ResNet50, ResNet101) initialized with ImageNet pre-trained weights. To adapt these general-purpose networks to the specific task of pituitary microadenoma detection on non-contrast MRI and to counteract overfitting, we instituted a dedicated preprocessing and optimization pipeline. All region-of-interest (ROI) patches were resized to 224 × 224 pixels. Image intensity underwent a two-stage normalization: initial min-max scaling to the [-1, 1] range to ensure consistent input value distribution, followed by dataset-wide Z-score standardization. During training, real-time data augmentation—incorporating random cropping and flipping—was applied to bolster model robustness and generalization. The model was optimized using stochastic gradient descent (SGD) with a softmax cross-entropy loss function, coupled with a cosine annealing learning rate scheduler to facilitate steady convergence. For the test images, the preprocessing pipeline was restricted to image resizing and intensity normalization, omitting augmentation steps.

### 2D_DL, 2.5D_DL and 2D multichannel model development and feature extraction

We developed three deep learning models based on the optimal ResNet architecture: a 2D model, a 2.5D model, and a 2D multichannel model. All models were initialized with pre-trained weights from ImageNet and fine-tuned on our dataset for 100 epochs. After fine-tuning, we extracted deep learning features from the penultimate layer (i.e., the global average pooling layer) of each model. This process yielded 512-dimensional feature vectors for each patient based on their tumor images. These extracted features served as input for subsequent analyses (Fig. S1-3). All feature extraction and model development were conducted on a single NVIDIA GeForce RTX 5080 16 GB GPU running Windows 10.

### Transformer model constructed based feature extraction

**Fig. 3:**
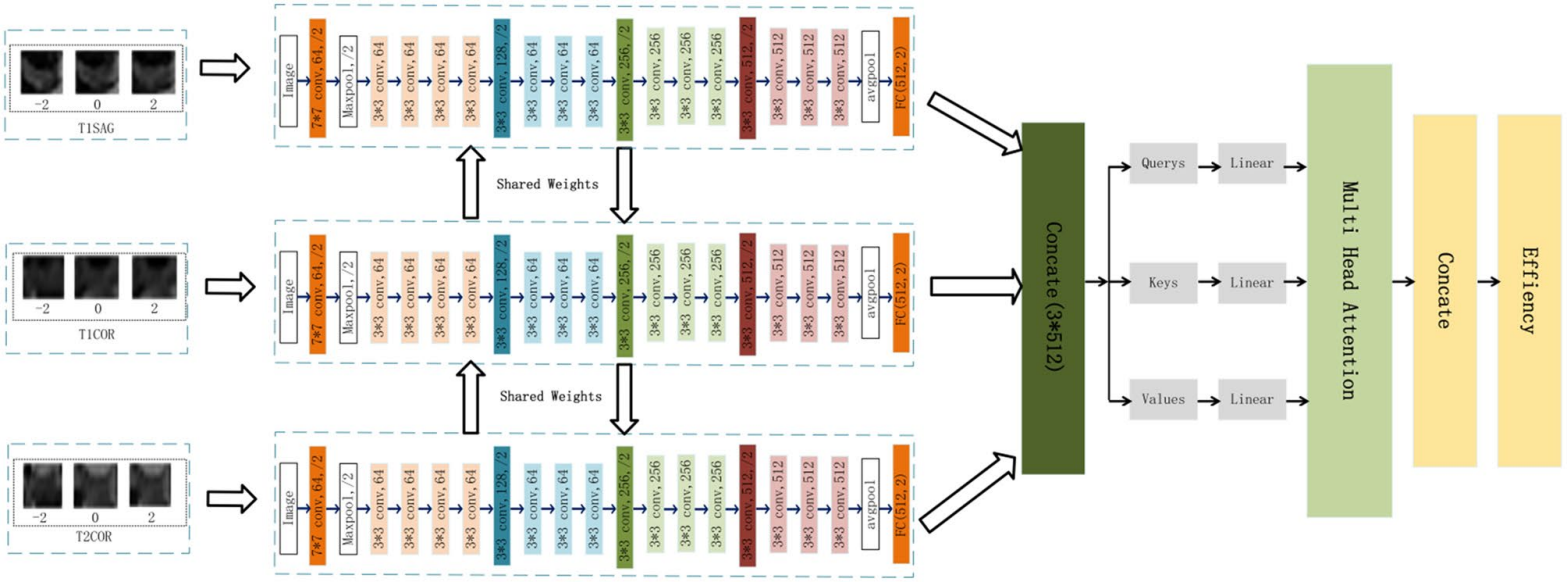
The Transformer model proposed in the study

We employ a standard Transformer encoder model (Fig. 3). The input is a sequence of three feature tokens (bags size = 3) extracted from multi-view MRI scans. Each token is embedded into a 512-dimensional space and added with sinusoidal positional encodings. The encoder consists of j = 6 identical layers, each containing a multi-head self-attention module with h = 8 heads and a position-wise feed-forward network. Residual connections and layer normalization (normalize=True) are applied after each sub-layer. The encoder outputs are aggregated via global average pooling for final prediction. The model was trained for 50 epochs using SGD with an initial learning rate of 0.001 and a batch size of 16, with a total training time of approximately 9 h and 52 min. To mitigate overfitting on the limited medical dataset, the ResNet-18 feature extractors were initialized with RadImageNet pre-trained weights and kept frozen throughout training. The checkpoint achieving the highest validation AUC—specifically at epoch 34—was retained as the final model for all subsequent testing. All experiments were conducted on a single NVIDIA GeForce RTX 5080 16 GB GPU running Windows 10.

### Evaluation of predictive efficacy

The evaluation metrics of different models’ prediction efficacy were calculated, including the accuracy, AUC, 95% CI, sensitivity, specificity, precision, recall, and F1-score. Radar chart was a graphical method of displaying multivariate data in the form of a two-dimensional chart. It is used to visually compare the skill profiles of these models, making it easy to see their relative strengths and weaknesses across AUC, specificity, precision, accuracy, recall, F1_score.

### Explainable AI using Grad-CAM and SHAP

Clinical translation of deep learning models requires more than high accuracy; it demands interpretability. To this end, we integrated two complementary explainable AI (XAI) techniques to elucidate how our model differentiates pituitary microadenomas. Gradient-weighted Class Activation Mapping (Grad-CAM) generates spatial heatmaps by computing the gradients of the target class score with respect to the feature maps of the final convolutional layer, effectively highlighting the regions in the input image that contributed most to the model’s prediction. Beyond spatial localization, we employed SHapley Additive exPlanations (SHAP) to dissect the contribution of individual features within our fused model. SHAP analysis assigns a quantitative value to each feature, precisely measuring its impact on shifting the prediction probability for a single case. The resultant force plots offer an intuitive, case-by-case breakdown, showing which features pushed the diagnosis toward or away from “microadenoma.”

Together, Grad-CAM and SHAP move the model from a black box toward a transparent decision-support tool, providing both visual and quantitative rationales aligned with clinical reasoning.

### Statistical analysis

Descriptive statistics present continuous variables as mean ± standard deviation for normally distributed data, and as frequencies (percentages) for categorical variables. Normality was assessed using the Shapiro-Wilk test. For group comparisons, we applied the independent-samples t-test or the Mann-Whitney U test to continuous variables, and the chi-square or Fisher’s exact test to categorical variables, choosing the test based on data characteristics. We examined associations between variables using Spearman’s rank correlation coefficient. Pairwise comparisons of AUCs between different models were performed using the DeLong test. To assess the added value of AI assistance, the diagnostic performance of each radiologist with and without model reference was compared. Sensitivity and specificity were compared using McNemar’s test for paired samples, and AUCs were compared using the DeLong test. A two-sided p-value below 0.05 was considered statistically significant. The statistical work was performed using SPSS (version 28.0), R (version 4.1.3), and Python (version 3.11.2).

## Results

### Participants baseline data

Our study included a total of 590 participants, with 321 cases in the training set, 137 cases in the internal validation and 132 cases in external validation (Table 1, S2).

**Table 1:** Baseline data on participants

			Center A				Center B		Center C	
			Training set	P	Val set	P	Test 1	P	Test 2	P
Age		Microadenoma	34.94 ± 12.51	0.272	38.03 ± 14.06	0.045	40.90 ± 15.66	0.069	37.05 ± 14.03	0.062
		Non-microadenoma	37.97 ± 18.41		32.14 ± 17.30		35.02 ± 16.94		25.67 ± 15.25	
Sex	Female	Microadenoma	140(81.40)	< 0.001	55(75.34)	0.029	41(78.85)	0.190	15(78.95)	0.014
		Non-microadenoma	84(56.38)		36(56.25)		34(65.38)		2(22.22)	
	Male	Microadenoma	32(18.60)		18(24.66)		11(21.15)		4(21.05)	
		Non-microadenoma	65(43.62)		28(43.75)		18(34.62)		7(77.78)	

**Table 2:** Performances of the 2D_DL predictive models in the study sets

Models name	Accuracy	AUC	95%CI	Sensitivity	Specificity	PPV	NPV	F1	Threshold	Cohort	*P* value* 2D_Multichannel vs.
2D_Multichannel	0.810	**0.893**	0.8592–0.9274	0.814	0.805	0.828	0.789	0.821	0.464	train	-
2D_Multichannel	0.766	**0.828**	0.7599–0.8968	0.973	0.531	0.703	0.944	0.816	0.456	val	-
2D_Multichannel	0.731	**0.773**	0.6835–0.8631	0.846	0.615	0.687	0.800	0.759	0.504	test1	-
2D_Multichannel	0.786	**0.743**	0.5470–0.9384	0.842	0.667	0.842	0.667	0.842	0.341	test2	-
2D_T1SAG	**0.798**	**0.884**	0.8496–0.9190	0.791	0.805	0.824	0.769	0.807	0.521	train	0.070
2D_T1SAG	0.745	0.818	0.7482–0.8871	0.712	0.781	0.788	0.704	0.748	0.646	val	0.792
2D_T1SAG	0.740	0.787	0.7002–0.8738	0.788	0.692	0.719	0.766	0.752	0.487	test1	0.810
2D_T1SAG	0.857	0.766	0.5044–0.9999	0.947	0.667	0.857	0.857	0.900	0.535	test2	0.883
2D_T1COR	0.723	0.779	0.7287–0.8285	0.698	0.752	0.764	0.683	0.729	0.495	train	< 0.005^***^
2D_T1COR	0.679	0.701	0.6143–0.7881	0.740	0.610	0.684	0.672	0.711	0.534	val	0.023^*^
2D_T1COR	0.673	0.687	0.5836–0.7887	0.846	0.500	0.629	0.765	0.721	0.362	test1	0.219
2D_T1COR	0.571	0.591	0.3611–0.8202	0.474	0.778	0.818	0.412	0.600	0.896	test2	0.318
2D_T2COR	0.763	0.846	0.8044–0.8868	0.657	0.886	0.869	0.710	0.759	0.609	train	0.070
2D_T2COR	0.679	0.745	0.6631–0.8267	0.548	0.828	0.784	0.616	0.645	0.883	val	0.073
2D_T2COR	0.592	0.582	0.4777–0.6995	0.569	0.615	0.592	0.593	0.580	0.923	test1	0.011^*^
2D_T2COR	0.714	0.596	0.3465–0.8464	0.842	0.444	0.762	0.571	0.800	0.657	test2	0.375

The p-values^*^ correspond to comparisons between the 2D_Multichannel model and each of the 2D models across all sets. AUC: the area under the ROC curve; 95% CI: 95% Confidence Interval. Test2 (*n*=28) has a limited sample size; its estimates should be interpreted with caution. *** *p* < 0.001, ** *p* < 0.01, * *p* < 0.05

**Table 3:** Performances of the 2.5D_DL predictive models in the study sets

Models name	Accuracy	AUC	95%CI	Sensitivity	Specificity	PPV	NPV	F1	Threshold	Cohort	*P* value* transformer vs.
2.5D_T1SAG	0.692	0.763	0.7114–0.8144	0.576	0.826	0.792	0.628	0.667	0.554	train	< 0.001^***^
2.5D_T1SAG	0.679	0.717	0.6311–0.8034	0.671	0.688	0.710	0.647	0.690	0.545	val	0.001^**^
2.5D_T1SAG	0.625	0.638	0.5307–0.7445	0.673	0.577	0.614	0.638	0.642	0.517	test1	0.002^**^
2.5D_T1SAG	0.679	0.725	0.5160–0.9343	0.579	0.889	0.917	0.500	0.710	0.641	test2	0.349
2.5D_T1COR	**0.807**	**0.863**	0.8225–0.9033	0.849	0.758	0.802	0.813	0.825	0.506	train	< 0.001^***^
2.5D_T1COR	0.693	0.676	0.5837–0.7682	0.781	0.594	0.687	0.704	0.791	0.572	val	< 0.001^***^
2.5D_T1COR	0.635	0.618	0.5079–0.7273	0.942	0.327	0.583	0.850	0.721	0.362	test1	0.001^**^
2.5D_T1COR	0.750	0.772	0.5698–0.9741	0.684	0.889	0.929	0.571	0.788	0.610	test2	0.757
2.5D_T2COR	0.757	0.835	0.7927–0.8782	0.715	0.805	0.809	0.710	0.759	0.609	train	< 0.001^***^
2.5D_T2COR	0.657	0.601	0.5045–0.6980	0.863	0.422	0.630	0.730	0.728	0.501	val	< 0.001^***^
2.5D_T2COR	0.553	0.500	0.3866–0.6127	0.647	0.462	0.541	0.571	0.589	0.655	test1	< 0.001^***^
2.5D_T2COR	0.714	0.520	0.2752–0.7658	0.947	0.222	0.720	0.667	0.818	0.386	test2	0.044^*^
transformer	0.944	**0.985**	0.9762–0.9945	0.919	0.973	0.975	0.912	0.946	0.540	train	-
transformer	0.796	**0.874**	0.8178–0.9305	0.795	0.797	0.812	0.773	0.806	0.540	val	-
transformer	0.779	**0.829**	0.7514–0.9069	0.923	0.635	0.716	0.892	0.807	0.424	test1	-
transformer	0.750	**0.819**	0.6151–0.9999	0.684	0.889	0.929	0.571	0.788	0.721	test2	-

The p-values^*^ correspond to comparisons between the transformer model and each of the 2.5D models across all sets. AUC: the area under the ROC curve; 95% CI: 95% Confidence Interval. Test2 (*n*=28) has a limited sample size; its estimates should be interpreted with caution. *** *p* < 0.001, ** *p* < 0.01, * *p* < 0.05

### Results of different deep learning algorithm at training and validation cohorts

Overall consideration, ResNet18 exhibited superior performance across diverse cohorts as evidenced by its AUC results in all series. Slice level prediction results are shown in Table S3-S5. Across all three validation sets, ResNet18 consistently achieves the highest or tied-highest Accuracy and AUC compared to the other models. While deeper networks like Resnet50 and Resnet101 sometimes perform better on the training set (e.g., T1SAG Resnet50 achieves a training AUC of 0.894), they show a significant performance drop on the validation set. Resnet18 exhibits the smallest gap between training and validation performance, suggesting it learns meaningful features without memorizing the training data, thus generalizing better to unseen data. The efficacy of ResNet18 in the present study underscores its utility for complex image recognition tasks, rendering it an exceptional option for extracting meaningful and discriminative features from medical images.

### Evaluation and comparison of the 2D and 2D_ResNet18_multichannel model

**Fig. 4:**
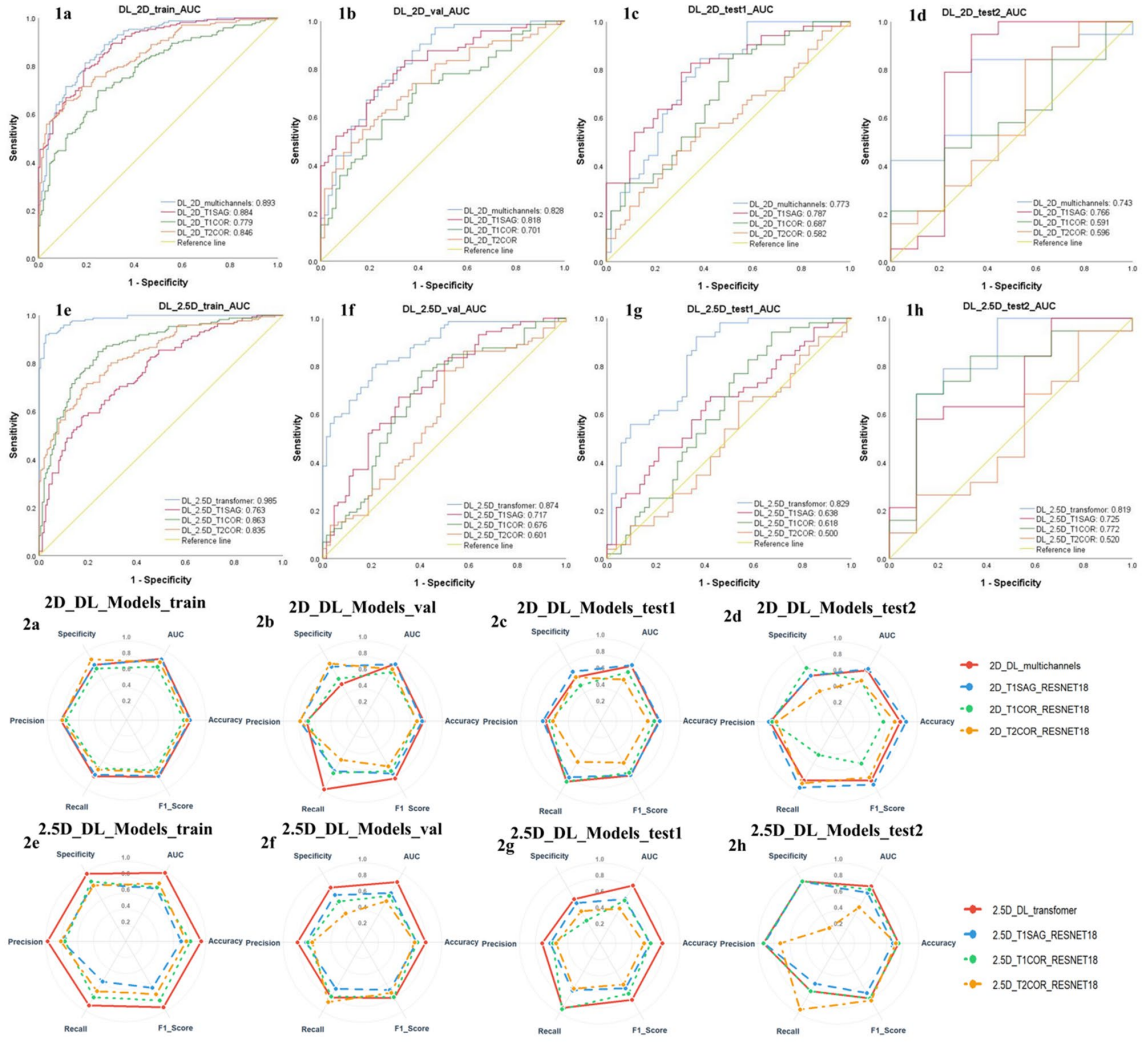
Performance comparison across datasets

The performances of the predictive models in the study sets were illustrated in Table 2; Fig. 4 and Fig. S4. The training performance of 2D_multichannel model was better than 2D_T1SAG_DL, 2D_T1COR_DL, 2D_T2COR_DL (AUC, 0.893 (95% Confidence Interval (CI): 0.8592–0.9274), 0.884 (95%CI: 0.8496–0.9190), 0.779 (95%CI: 0.7287–0.8285), and 0.846 (95%CI: 0.8044–0.8868), respectively). Single-sequence models showed considerable performance fluctuation across different datasets. For instance, while the T2COR model performed well on the training set (AUC 0.846), its performance dropped sharply on Test1 (AUC 0.582), indicating vulnerability to changes in data distribution. In contrast, the Multichannel model maintained relatively stable and high performance across all datasets (Train, Val, Test1, Test2) with AUCs of 0.893, 0.828, 0.773, and 0.743 respectively, demonstrating its superior robustness and reliability. Compared to the weakest performer (T1COR), the improvement of the Multichannel model was statistically significant in both the training (*P* < 0.005) and validation (*P* = 0.023) cohorts. This confirms that fusing multi-sequence information yields a tangible performance gain over relying on a single coronal T1 sequence.

### Evaluation and comparison of the 2.5D_ResNet18_DL and transformer model

The performances of the predictive models in the study sets were illustrated in Table 3; Fig. 4 and Fig. S5. The AUC of each single sequence DL model (2.5D_T1SAG, 2.5D_T1COR and 2.5D_T2COR) was 0.763 (95% CI: 0.7714–0.8144), 0863 (95% CI: 0.8225–0.9033), 0.835 (95% CI: 0.7927–0.8782) in the training cohort, respectively. The 2.5D_T1COR model achieved the highest accuracy 0.807. Crucially, in the training set, the Transformer model achieved an AUC (0.985) and Accuracy (0.944) far exceeding all 2.5D single-sequence models (T1SAG, T1COR, T2COR with AUCs of 0.763, 0.863, and 0.835, respectively), and this advantage is highly statistically significant (*P* < 0.001) compared to each single-sequence model. The Transformer not only excelled on the training set but also maintained remarkably high diagnostic performance on the validation set, Test1, and Test2 (AUCs of 0.874, 0.829, and 0.819, respectively). This demonstrates its superior feature extraction capabilities and excellent generalization, achieved the best and most stable performance across all datasets. It stands as the optimal model architecture for processing this type of 2.5D medical imaging data.

### Comparison of model predictive performance using AUC and radar charts

Figure 4 illustrates the above results and shows that the 2.5D_transformer model consistently achieved higher AUC values and the superior predictive capability across all datasets for accurately identifying the presence of pituitary microadenomas. The performance of all models was further evaluated using calibration curves and decision curve analysis (DCA) across the training, validation, and test sets, as illustrated in Figure S6.

### Model interpretability via SHAP and Grad-CAM

**Fig. 5:**
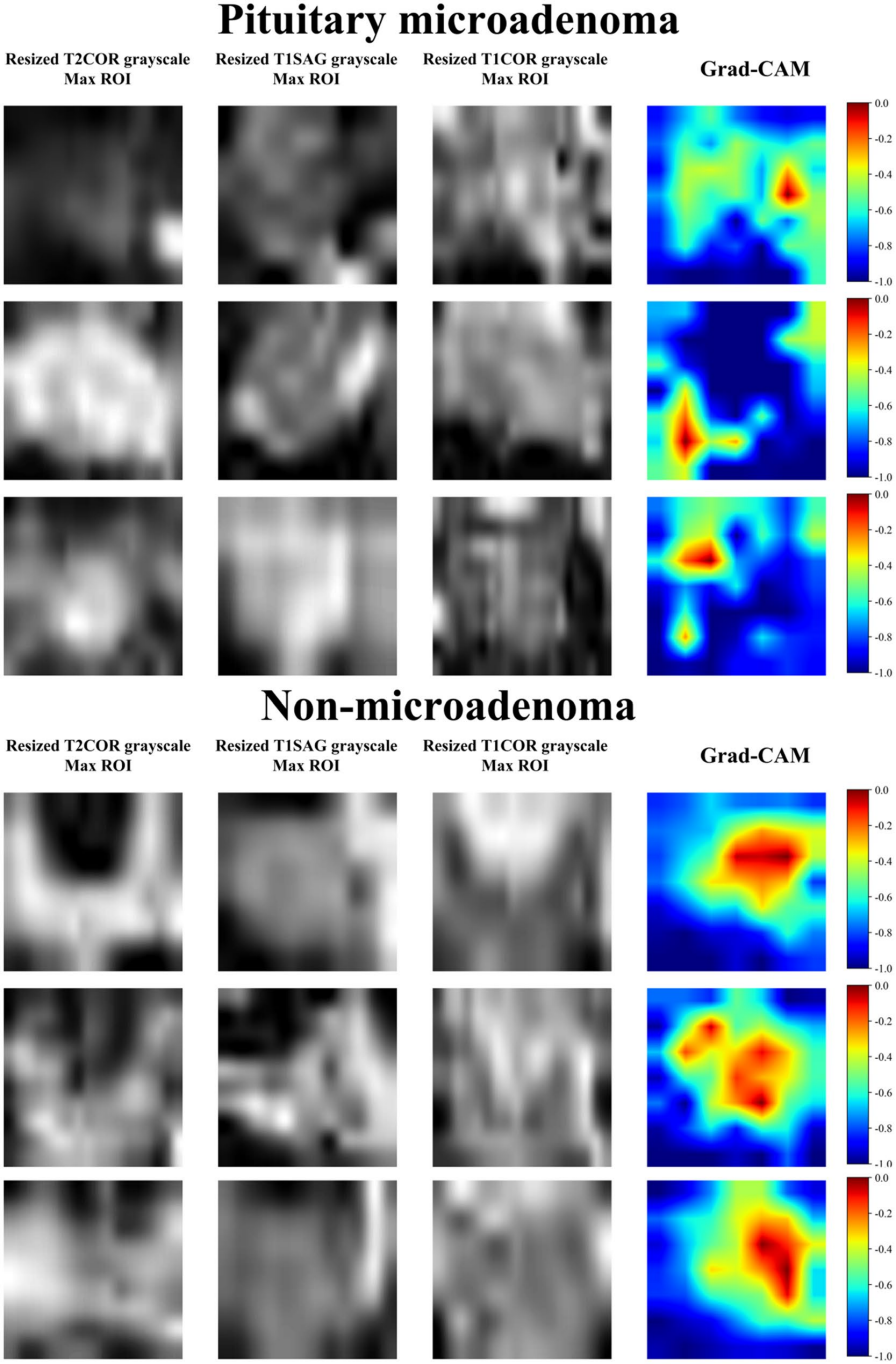
Examples of Grad-CAM visualization generated by our multichannel model for pituitary microadenoma classification. The three grayscale images (resized T2COR, TISAG, and TICOR ROIs) were stacked as multichannel inputs to the model. For the microadenoma case (top), the Grad-CAM accurately highlights a focal region within the sella turcica corresponding to the lesion. For the non-microadenoma case (bottom), the Grad-CAM shows diffuse activation across the normal pituitary gland, indicating the absence of localized pathological features

**Fig. 6:**
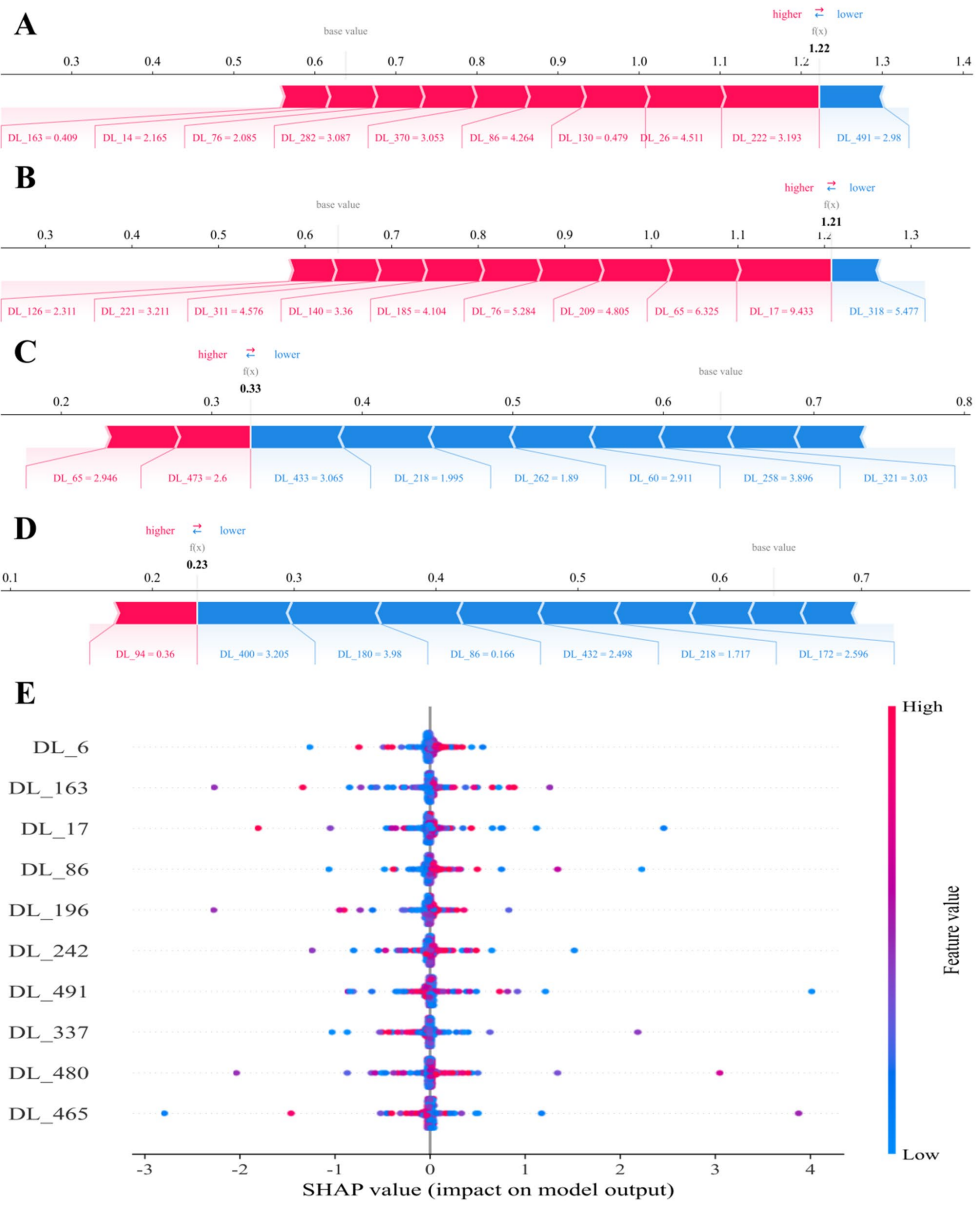
SHAP force plots for local interpretation of predictions about transformer model. Blue segments (left-pointing arrows) indicate features reducing prediction risk (negative SHAP values), while red segments (right-pointing arrows) denote features increasing risk (positive SHAP values). The baseline value [f(x)] represents the model’s mean prediction. Four representative cases demonstrate: (**A**-**B**) two pituitary microadenoma cases with high predicted risk (88.5% and 98.4%); (**C**-**D**) two healthy cases from physical/preconception examinations with low predicted risk (24.6% and 5.5%). (**E**) SHAP summary plot displaying the impact and importance of deep learning features on model predictions, with color indicating feature values

Some examples are shown in Fig. 5. For the microadenoma case, the Grad-CAM of 2D_multichannel model accurately highlights a focal region within the sella turcica corresponding to the lesion. For the non-microadenoma case, the Grad-CAM shows diffuse activation across the normal pituitary gland, indicating the absence of localized pathological features. And to further enhance the interpretability of the 2.5D_DL transformer model, Fig. 6a-b illustrates a patient with a SHAP value of 1.22 and 1.21 (> 0.5 baseline), classifying them into the high predictive risk group. In contrast, Fig. 6c-d demonstrates a patient with a SHAP value of 0.33 and 0.23 (< 0.5 baseline), indicative of low predictive risk group.

### Radiologist performance with and without AI assistance

We evaluated the diagnostic performance of three independent radiologists (Radiogist1, Radiogist2, Radiogist3) with varying levels of experience, both unaided and with reference to two deep learning models (2D_Multichannel and 2.5D_Transformer), on the internal test set. The detailed performance metrics are presented in Tables 4 and 5. Without AI assistance, all three radiologists demonstrated moderate and comparable diagnostic performance. The AUC values ranged from 0.520 to 0.541, with corresponding accuracies between 0.461 and 0.520. Sensitivities and specificities were similarly suboptimal, ranging from 0.486 to 0.528 and 0.432 to 0.512, respectively.

**Table 4:** Classification performance of radiologists with and without the 2D_Multichannel model as a reference in the testing sets

Variable	AUC(95%CI)	ACC	SEN	SPE	PPV	NPV	*P*-value
Radiologist 1	0.522 (0.462–0.582)	0.480	0.500	0.456	0.514	0.442	< 0.001***
Radiologist 2	0.541 (0.481–0.601)	0.461	0.486	0.432	0.497	0.422	< 0.001***
Radiologist 3	0.520 (0.460–0.580)	0.520	0.528	0.512	0.555	0.485	< 0.001***
Radiologist 1 with 2D_Multichannel	0.725 (0.674–0.777)	0.736	0.875	0.576	0.704	0.800	< 0.001***
Radiologist 2 with 2D_Multichannel	0.716 (0.661–0.770)	0.718	0.743	0.688	0.733	0.700	0.040*
Radiologist 3 with 2D_Multichannel	0.755 (0.703–0.806)	0.758	0.806	0.704	0.758	0.759	0.421

The P-values correspond to comparisons between the 2D_Multichannel model with each radiologist, both with and without the model as a reference. ACC accuracy, SEN sensitivity, SPE specificity, PPV positive predictive value, NPV negative predictive value, 95% CI 95% confidence interval. *** *p* < 0.001, ** *p* < 0.01, * *p* < 0.05

**Table 5:** Classification performance of radiologists with and without the 2.5D_transformer model as a reference in the testing sets

Variable	AUC(95%CI)	ACC	SEN	SPE	PPV	NPV	*P*-value
Radiologist 1	0.522 (0.462–0.582)	0.480	0.500	0.456	0.514	0.442	< 0.001***
Radiologist 2	0.541 (0.481–0.601)	0.461	0.486	0.432	0.497	0.422	< 0.001***
Radiologist 3	0.520 (0.460–0.580)	0.520	0.528	0.512	0.555	0.485	< 0.001***
Radiologist 1 with 2.5D_transformer	0.710 (0.658–0.762)	0.721	0.868	0.552	0.691	0.784	< 0.001***
Radiologist 2 with 2.5D_transformer	0.672 (0.615–0.728)	0.673	0.688	0.656	0.692	0.646	< 0.001***
Radiologist 3 with 2.5D_transformer	0.737 (0.684–0.790)	0.740	0.771	0.704	0.750	0.727	< 0.001***

The P-values correspond to comparisons between the 2.5D_transformer model with each radiologist, both with and without the model as a reference. ACC accuracy, SEN sensitivity, SPE specificity, PPV positive predictive value, NPV negative predictive value, 95% CI 95% confidence interval. *** *p* < 0.001, ** *p* < 0.01, * *p* < 0.05

When the 2D_Multichannel model was provided as a reference, the diagnostic performance of all radiologists improved substantially. The AUCs increased significantly to a range of 0.716–0.755, representing an absolute improvement of approximately 0.20 compared to unaided reads. Notably, Radiologist 3 achieved the highest AUC (0.755; 95% CI: 0.703–0.806) and accuracy (0.758) when assisted by this model. The improvements were comprehensive across all metrics: sensitivity increased up to 0.875 (Radiologist 1) and specificity up to 0.704 (Radiologist 3). All improvements were statistically significant (*P* < 0.05), with the exception of Radiologist 3 (*P* = 0.421), which may be attributed to his/her relatively higher baseline performance or limited sample size.

Similarly, the 2.5D_Transformer model significantly enhanced radiologists’ diagnostic performance (all *P* < 0.001), with AUCs improving to a range of 0.672–0.737. Radiologist 3 again demonstrated the highest AUC (0.737; 95% CI: 0.684–0.790) and accuracy (0.740) with model assistance. Sensitivity improved markedly, reaching up to 0.868 (Radiologist 1), while specificity reached up to 0.704 (Radiologist 3).

## Discussion

We developed a 2.5D Transformer model to detect pituitary microadenomas on conventional, non-contrast multiparametric MRI. By integrating different MRI sequences and orientations as distinct input, our model achieved satisfactory classification performance with AUCs of 0.985 and 0.874 on the training and validation sets. These results confirm that multi-sequence MRI data, even without contrast, carries substantial diagnostic information. This is likely due to the complementary nature of multi-sequence imaging, which provides both information redundancy and feature diversity, thereby facilitating effective data fusion and synergistic interpretation. To our knowledge, this is the first study to systematically compare 2D, 2.5D, and Transformer-based models for microadenoma detection on non-contrast MRI. Through large-scale, multi-center validation, we establish an empirical benchmark for this clinically challenging task. Our findings suggest that AI-augmented non-contrast MRI protocols could, in time, offer a viable alternative to contrast-enhanced imaging in routine clinical practice.

As demonstrated by our results, the proposed 2.5D Transformer model consistently outperformed all other models, not only on the training set but also on the internal validation and two external test sets. This indicates that it possesses sufficient robustness in practical applications. Admittedly, the Transformer model exhibits notable differences in performance (AUC 0.829 vs. 0.819) and optimal thresholds (0.424 vs. 0.721) across different test sets. This finding aligns closely with the theoretical prediction recently proposed by Kwon et al. [33]: If the pre-training tasks are drawn from a single Gaussian, the test risk shows a non-negligible dependence on the angle, implying that in-Context learning cannot generalize out-of-distribution. when test data deviates from training data due to “angular bias,” the test risk of Transformers exhibits a dependence on the direction of the shift. Our empirical results precisely validate this theoretical insight: the substantially different optimal thresholds required by the two external test sets (0.424 vs. 0.721) reflect distinct shift directions, while the stepwise performance degradation from the training set (0.985) to the validation set (0.874) and further to the test sets (0.82–0.83) demonstrates a dependence on shift magnitude. Notably, it is precisely because we selected the best model based on validation performance, rather than an overfitted final model, that the true effect of this angular bias is accurately revealed, rather than being obscured by overfitting noise.

We further employed Grad-CAM and SHAP to visualize model decisions. Integrating these models as reference tools significantly improved radiologists’ diagnostic performance: with the 2D_Multichannel model, AUCs rose from 0.520 to 0.541 to 0.716–0.755; with the 2.5D_Transformer, to 0.672–0.737 (Tables S6–S7). Interestingly, although the Transformer outperformed the 2D_Multichannel model in standalone tests (AUC: 0.829 vs. 0.773), it yielded smaller and less consistent collaborative gains. This suggests that algorithmic superiority does not automatically translate to effective clinical decision support—simply presenting a complex model’s prediction may not maximize human-AI synergy. Bridging this “algorithm-to-clinical” gap through optimized interfaces and explainability strategies remains a key direction for future work.

Nevertheless, several limitations of this study should be noted. First, the study is subject to potential selection bias due to its retrospective design and the fact that all data were collected from a single country. This may result in overrepresentation of certain disease patterns or population-specific characteristics. Future studies could mitigate this bias by incorporating more diverse multinational and multi-center data to improve sample diversity and representativeness, therefor to a wider spectrum of shift directions during training, potentially enhancing its robustness and reducing the need for site-specific threshold tuning. Second, the imaging data were acquired using equipment from Siemens Health manufacturers with relatively homogeneous acquisition protocols and resolutions. To enhance the generalizability and robustness of the model, future work should involve extensive data harmonization and model fine-tuning using images from a wider range of devices (GE, PHILIPS, and United Imaging Healthcare, et al.) and imaging settings. Third, the current pipeline relies on manual ROI segmentation by radiologists, which limits its applicability in fully automated clinical workflows. While we demonstrated high inter-rater consistency (ICC > 0.75), future work should focus on developing automated segmentation methods—such as detection-segmentation cascade networks or segmentation-diagnosis joint optimization—to eliminate this dependency and facilitate seamless clinical integration.

## Conclusion

In summary, we have developed a high-performance classifier for pituitary microadenomas by integrating 2.5D deep learning features derived from non-contrast, multiparametric MRI. This approach, enhanced by explainable AI techniques, provides not only a gain in diagnostic accuracy but also a transparent decision-support framework with the potential to meaningfully inform clinical workflow. The combination of XAI techniques, such as Grad-CAM and SHAP, enhances the interpretability and reliability of the system. High predictive accuracy and open-minded decision-making is the synergy that makes the model potentially an important clinical decision support tool. Based on such positive findings, future research should focus on addressing the current limitations, particularly through extensive clinical validation, to fully translate the benefits of this innovative tool into routine practice.

## Electronic Supplementary Material

Below is the link to the electronic supplementary material.

Supplementary Material 1

## Data Availability

The datasets used and/or analysed during the current study are available from the corresponding author on reasonable request.

## Abbreviations

MRI: Magnetic Resonance Imaging
DL: Deep learning
CNN: Convolutional neural network
XAI: Explainable AI
SHAP: SHapley Additive exPlanations
AUC: Area under the curve
PitNET: Pituitary neuroendocrine tumors
DCE-MRA: Dynamic contrast-enhanced magnetic resonance imaging
AI: Artificial intelligence
Grad-CAM: Gradient-weighted Class Activation Mapping
PACS: Picture archiving and communication system
DICOM: Digital imaging and communications in medicine
95%CI: 95% Confidence Interval
