# Supplementary material for: A multicenter-validated interpretable transformer model for pituitary microadenoma detection on non-contrast multiparametric MRI
Source: BMC Med Imaging. 2026 May 2;26:305. doi: 10.1186/s12880-026-02391-3 (PMC13288575; doi:10.1186/s12880-026-02391-3)

**ELECTRONIC SUPPLEMENTARY MATERIAL**

**Part I**. Supplementary Tables

**Part II**. Supplementary Figures

Table S1. The parameter details of primary sequences.

|  | Series | Repetition time(ms) | Echo time(ms) | Field of view(mm^2^) | Slice thickness(mm) | Voxel size(mm) | Affiliated unit |
| --- | --- | --- | --- | --- | --- | --- | --- |
| Siemens Verio  3.0T | T1WI COR | 2000 | 9.1 | 200×200 | 2.5 | 1.0×0.8×2.5 | Center A |
|  | T2WI COR | 4000 | 94 | 200×200 | 2.5 | 0.8×0.6×2.5 |  |
|  | T1WI SAG | 2000 | 9.1 | 200×200 | 2.5 | 0.8×0.6×2.5 |  |
| Siemens Aera  1.5T | T1WI COR | 2000 | 7.2 | 180×180 | 3.0 | 0.7×0.7×3.0 | Center A |
|  | T2WI COR | 2500 | 77 | 200×200 | 3.0 | 0.6×0.6×3.0 |  |
|  | T1WI SAG | 2200 | 8.3 | 160×160 | 3.0 | 0.6×0.6×3.0 |  |
| Siemens Avanto 1.5T | T1WI COR | 262 | 9.1 | 240×240 | 2.5 | 1.1×0.9×2.5 | Center B |
|  | T2WI COR | 2800 | 88 | 210×210 | 2.5 | 0.8×0.8×2.5 |  |
|  | T1WI SAG | 324 | 17 | 190×190 | 2.5 | 0.7×0.7×2.5 |  |
| Siemens Avanto 1.5T | T1WI COR | 742 | 14 | 200×200 | 2.0 | 0.4×0.4×2.0 | Center C |
|  | T2WI COR | 2900 | 30 | 200×200 | 2.0 | 0.6×0.6×2.0 |  |
|  | T1WI SAG | 563 | 10 | 200×200 | 2.0 | 0.4×0.4×2.0 |  |

T2WI, T2-weighted imaging; T1WI, T1-weighted imaging; COR, Coronal position; SAG, Sagittal position.

Table S2. Patient characteristics among different cohorts

|  | | Training set | Val set | Test 1 | Test 2 | P |
| --- | --- | --- | --- | --- | --- | --- |
| Age | | 34.35±15.58 | 35.28±15.87 | 37.96±16.50 | 33.39±15.15 | 0.446 |
| sex | Female | 224(69.78) | 91(66.42) | 75(72.12) | 17(60.71) | 0.596 |
|  | Male | 97(30.22) | 46(33.58) | 29(27.88) | 11 (39.29) |  |

Table. S3 Comparison of the performance of different Deep Learning models based on T1SAG slice level predictions.

|  | Accuracy | AUC | 95%CI | Sensitivity | Specificity | PPV | NPV | cohort |
| --- | --- | --- | --- | --- | --- | --- | --- | --- |
| 2D_Resnet18 | 0.798 | 0.884 | 0.8496-0.9190 | 0.791 | 0.805 | 0.824 | 0.769 | train |
| 2D_Resnet101 | 0.782 | 0.866 | 0.8282-0.9038 | 0.733 | 0.839 | 0.840 | 0.731 | train |
| 2D_Resnet50 | 0.816 | 0.894 | 0.8605-0.9267 | 0.820 | 0.812 | 0.834 | 0.796 | train |
| 2D_Resnet18 | 0.745 | 0.818 | 0.7482-0.8871 | 0.712 | 0.781 | 0.788 | 0.705 | val |
| 2D_Resnet101 | 0.674 | 0.705 | 0.6156-0.7939 | 0.592 | 0.776 | 0.764 | 0.608 | val |
| 2D_Resnet50 | 0.698 | 0.715 | 0.6271-0.8037 | 0.690 | 0.707 | 0.742 | 0.651 | val |
| 2.5D_Resnet18 | 0.692 | 0.763 | 0.714-0.8144 | 0.576 | 0.826 | 0.792 | 0.628 | train |
| 2.5D_Resnet101 | 0.614 | 0.619 | 0.5570-0.6801 | 0.797 | 0.403 | 0.606 | 0.632 | train |
| 2.5D_Resnet50 | 0.636 | 0.668 | 0.6094-0.7271 | 0.558 | 0.725 | 0.701 | 0.587 | train |
| 2.5D_Resnet18 | 0.679 | 0.717 | 0.6311-0.8064 | 0.671 | 0.688 | 0.710 | 0.647 | val |
| 2.5D_Resnet101 | 0.597 | 0.540 | 0.4383-0.6419 | 0.761 | 0.397 | 0.607 | 0.575 | val |
| 2.5D_Resnet50 | 0.628 | 0.604 | 0.5058-0.7030 | 0.620 | 0.638 | 0.677 | 0.578 | val |

Table. S4 Comparison of the performance of different Deep Learning models based on T1COR slice level predictions.

|  | Accuracy | AUC | 95%CI | Sensitivity | Specificity | PPV | NPV | Cohort |
| --- | --- | --- | --- | --- | --- | --- | --- | --- |
| 2D_Resnet18 | 0.723 | 0.779 | 0.7287-0.8285 | 0.678 | 0.752 | 0.764 | 0.683 | Train |
| 2D_Resnet101 | 0.766 | 0.838 | 0.7959-0.8811 | 0.721 | 0.819 | 0.821 | 0.718 | Train |
| 2D_Resnet50 | 0.682 | 0.746 | 0.6927-0.7986 | 0.547 | 0.839 | 0.797 | 0.616 | Train |
| 2D_Resnet18 | 0.679 | 0.701 | 0.6143-0.7881 | 0.740 | 0.610 | 0.684 | 0.672 | Val |
| 2D_Resnet101 | 0.659 | 0.645 | 0.5494-0.7411 | 0.690 | 0.621 | 0.690 | 0.621 | Val |
| 2D_Resnet50 | 0.651 | 0.671 | 0.5783-0.7636 | 0.648 | 0.655 | 0.697 | 0.603 | Val |
| 2.5D_Resnet18 | 0.807 | 0.863 | 0.8225-0.9033 | 0.849 | 0.758 | 0.802 | 0.813 | Train |
| 2.5D_Resnet101 | 0.564 | 0.521 | 0.4570-0.5845 | 0.895 | 0.181 | 0.558 | 0.600 | Train |
| 2.5D_Resnet50 | 0.623 | 0.610 | 0.5481-0.6724 | 0.866 | 0.342 | 0.603 | 0.689 | Train |
| 2.5D_Resnet18 | 0.693 | 0.676 | 0.5837-0.7682 | 0.781 | 0.594 | 0.687 | 0.704 | Val |
| 2.5D_Resnet101 | 0.550 | 0.500 | 1.000-1.000 | 1.000 | 0.000 | 0.550 | 0.000 | Val |
| 2.5D_Resnet50 | 0.636 | 0.570 | 0.4678-0.6726 | 0.887 | 0.328 | 0.618 | 0.704 | Val |

Table. S5 Comparison of the performance of different Deep Learning models based on T2COR slice level predictions.

|  | Accuracy | AUC | 95%CI | Sensitivity | Specificity | PPV | NPV | Cohort |
| --- | --- | --- | --- | --- | --- | --- | --- | --- |
| 2D_Resnet18 | 0.763 | 0.846 | 0.8044-0.8868 | 0.657 | 0.886 | 0.869 | 0.691 | Train |
| 2D_Resnet101 | 0.760 | 0.822 | 0.7775-0.8667 | 0.744 | 0.779 | 0.795 | 0.725 | Train |
| 2D_Resnet50 | 0.763 | 0.844 | 0.8018-0.8855 | 0.680 | 0.859 | 0.848 | 0.699 | Train |
| 2D_Resnet18 | 0.679 | 0.745 | 0.6631-0.8267 | 0.548 | 0.828 | 0.784 | 0.616 | Val |
| 2D_Resnet101 | 0.667 | 0.643 | 0.5471-0.7395 | 0.817 | 0.483 | 0.660 | 0.683 | Val |
| 2D_Resnet50 | 0.659 | 0.645 | 0.5485-0.7414 | 0.704 | 0.603 | 0.685 | 0.625 | Val |
| 2.5D_Resnet18 | 0.757 | 0.835 | 0.7927-0.8782 | 0.715 | 0.805 | 0.809 | 0.710 | Train |
| 2.5D_Resnet101 | 0.592 | 0.581 | 0.5179-0.6432 | 0.767 | 0.356 | 0.588 | 0.602 | Train |
| 2.5D_Resnet50 | 0.526 | 0.518 | 0.4543-0.5810 | 0.244 | 0.852 | 0.656 | 0.494 | Train |
| 2.5D_Resnet18 | 0.657 | 0.601 | 0.5045-0.6980 | 0.863 | 0.422 | 0.630 | 0.730 | Val |
| 2.5D_Resnet101 | 0.543 | 0.506 | 0.4046-0.6065 | 0.535 | 0.552 | 0.594 | 0.492 | Val |
| 2.5D_Resnet50 | 0.550 | 0.500 | 1.0000-1.0000 | 1.000 | 0.000 | 0.550 | 0.000 | Val |

Fig. S1. Architecture of 2D ResNet18 model proposed in the study.


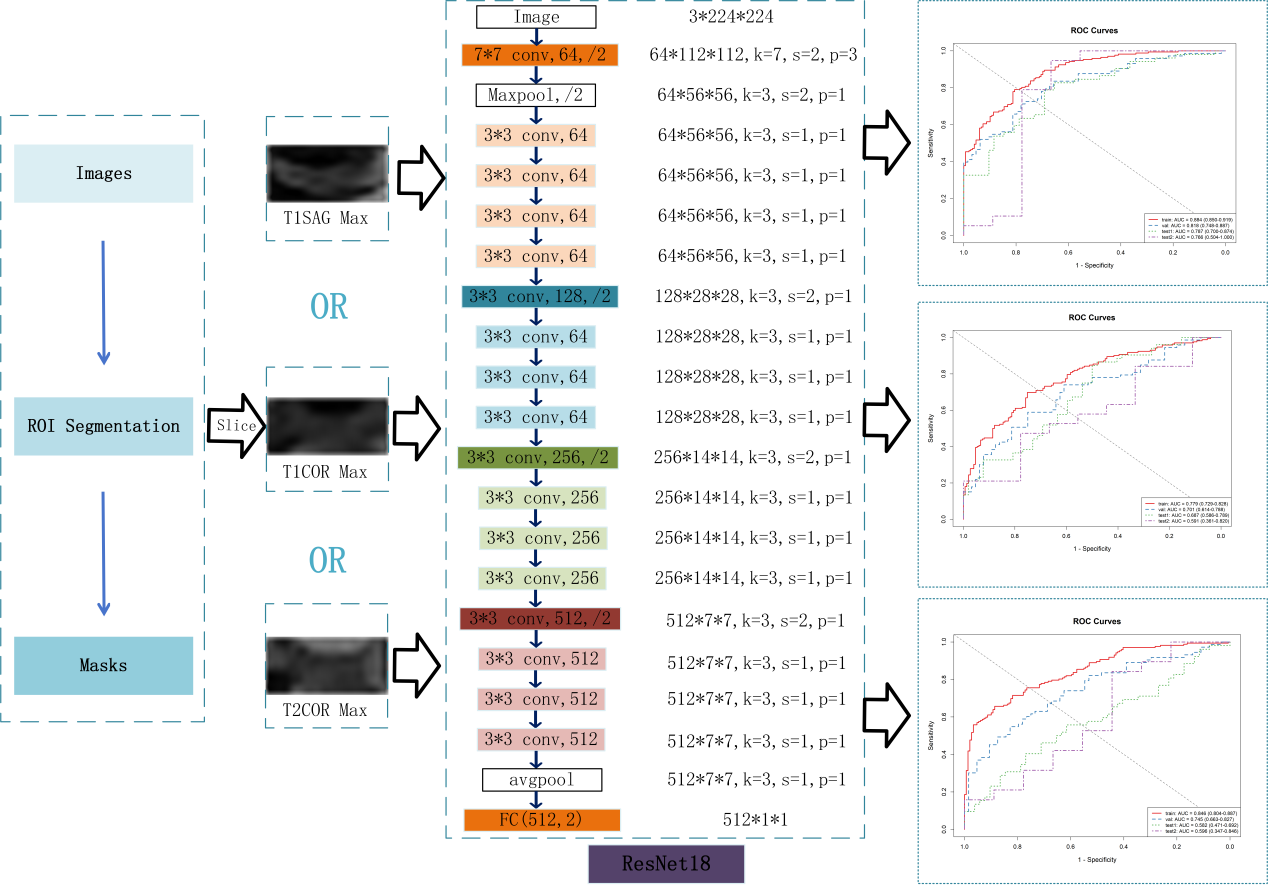


Fig. S2. Architecture of 2.5D ResNet18 model proposed in the study.


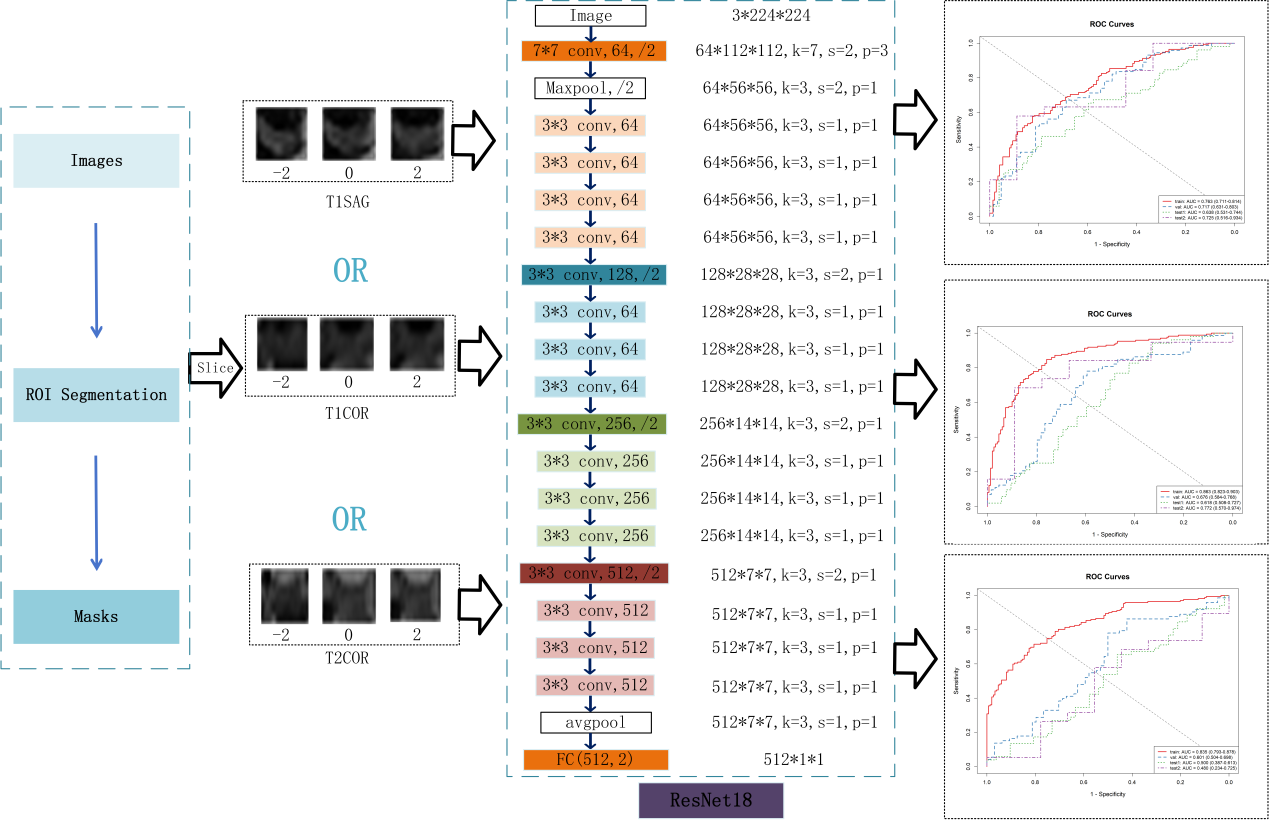


Fig. S3. Architecture of 2D ResNet18 multichannel model proposed in the study.


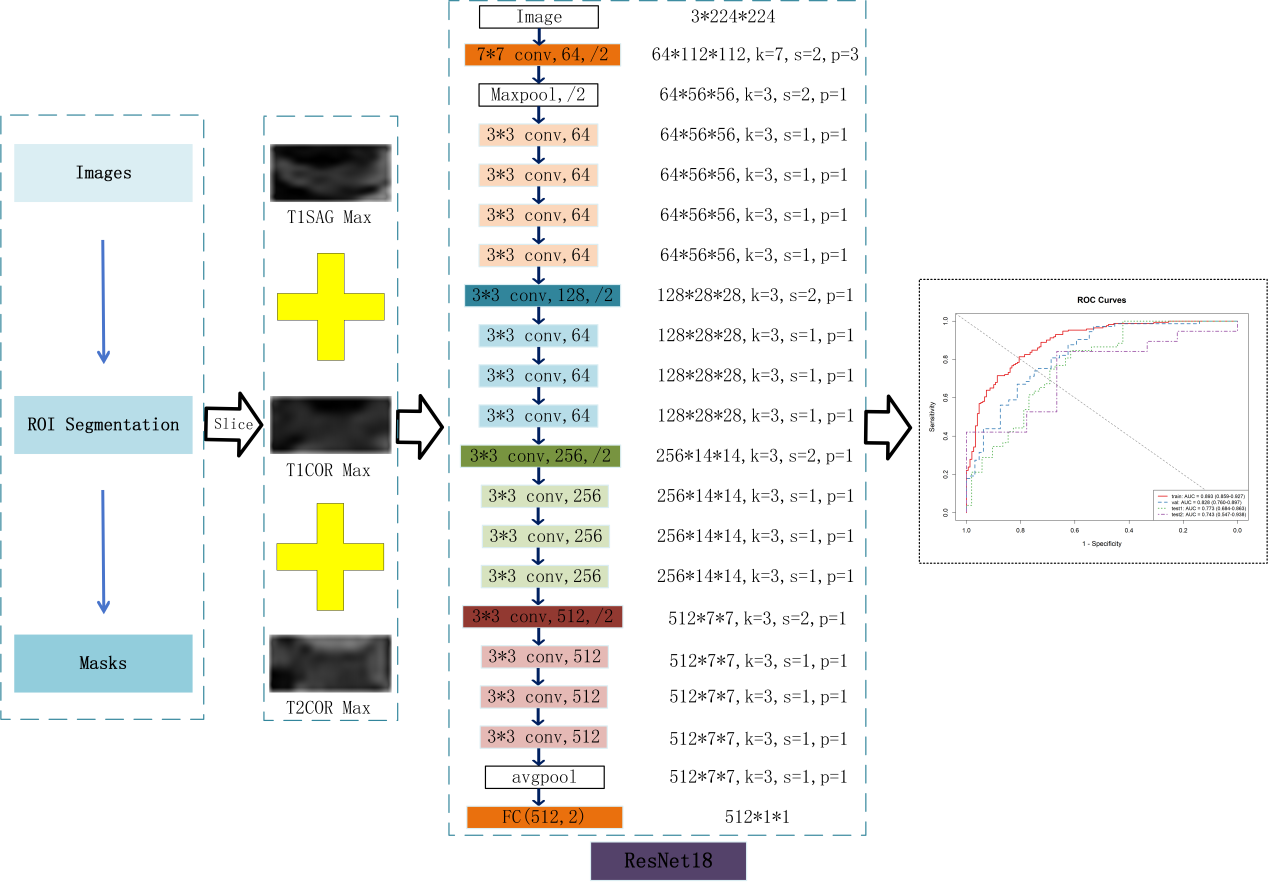


Figure S4. Diagnostic performance of the 2D_DL models across all datasets.

*** p < 0.001, ** p < 0.01, * p < 0.05, ns: No significant difference.


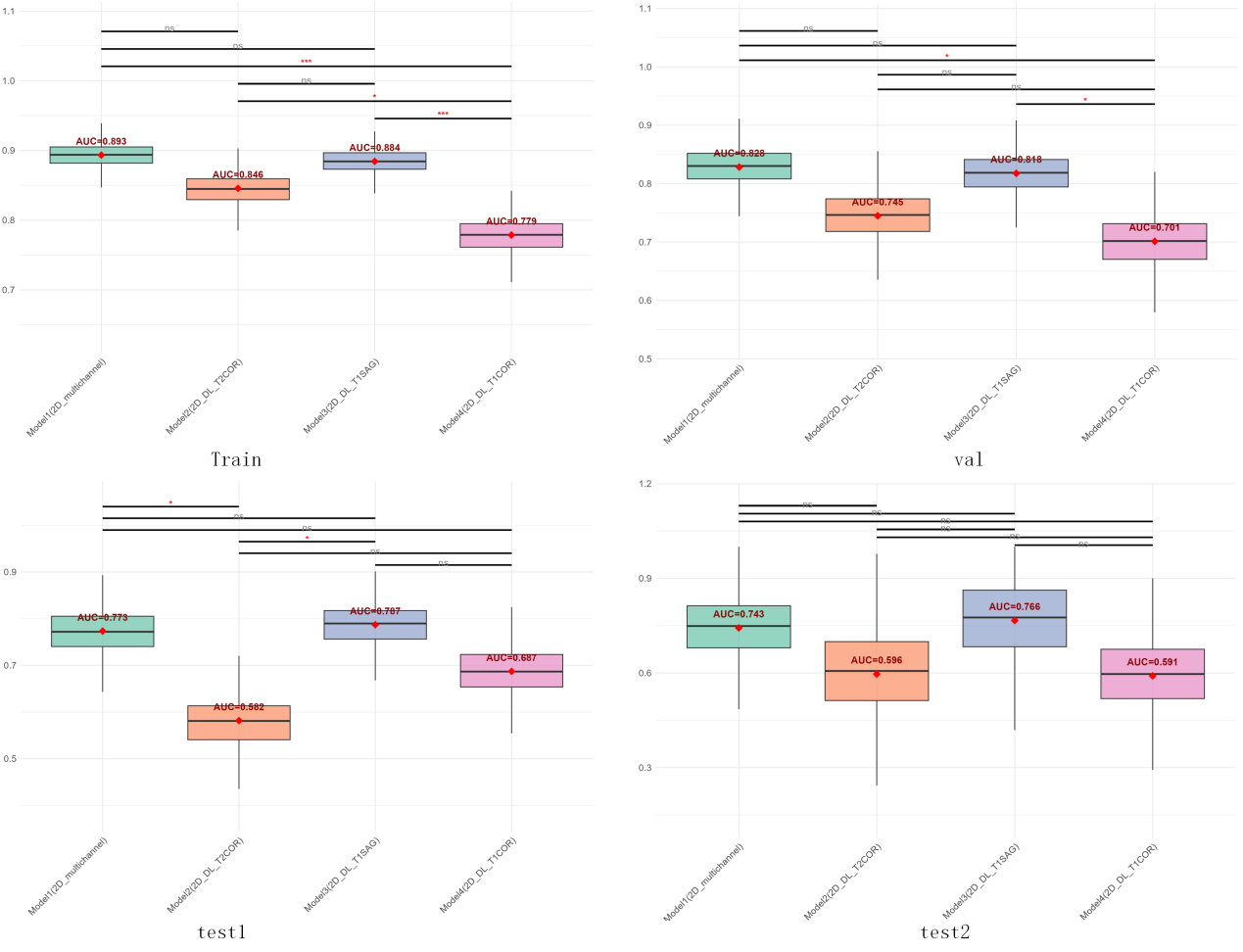


Figure S5. Diagnostic performance of the 2.5D_DL models across all datasets. *** p < 0.001, ** p < 0.01, * p < 0.05, ns: No significant difference.


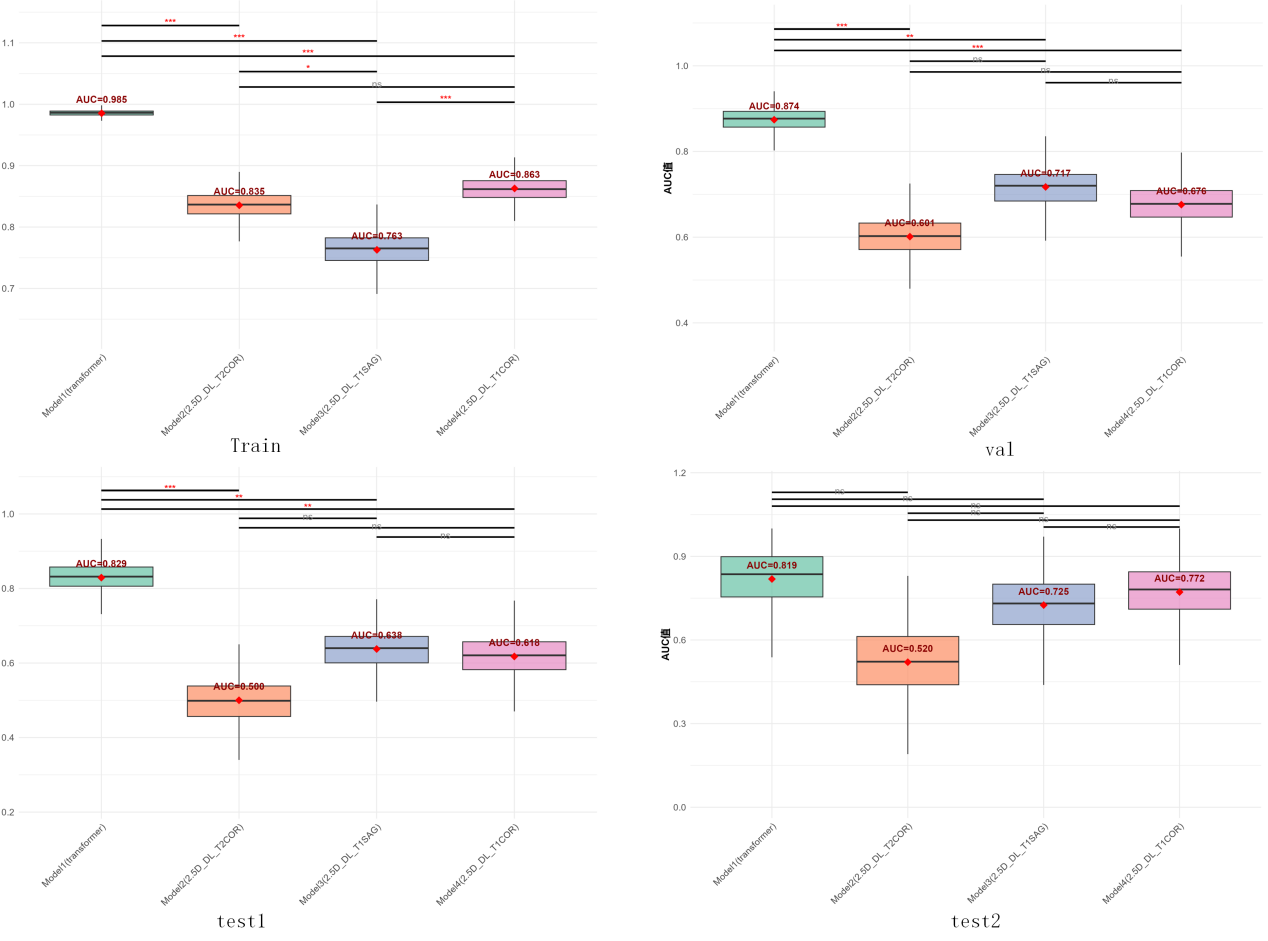


**Figure S6. Calibration curves and decision curve analysis of all models in the train, validation and test sets.**


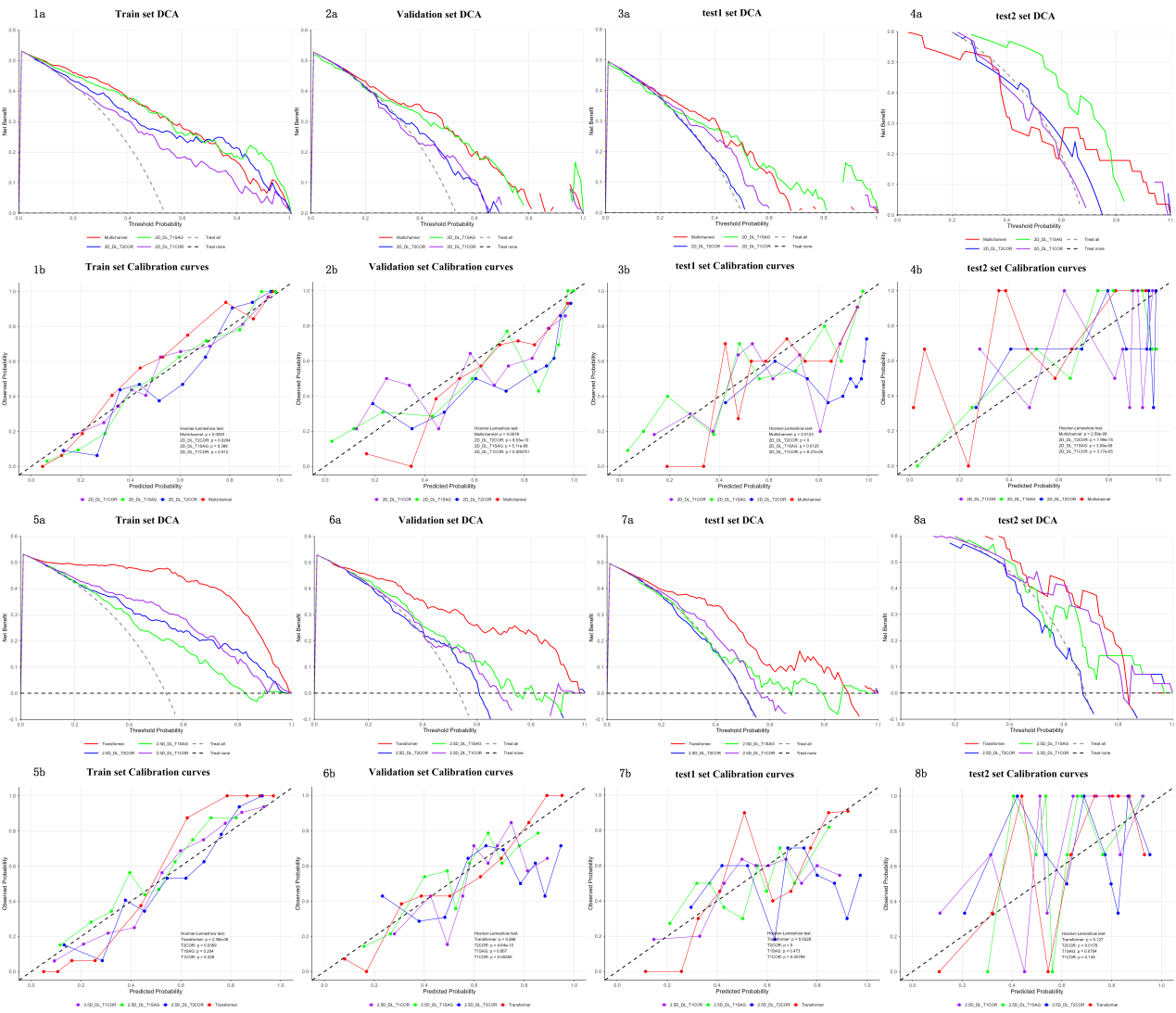

Supplement: Supplementary file 1 — Supplementary Material 1 [file 12880_2026_2391_MOESM1_ESM.docx]
